# Identification and Characterization of Long Non-Coding RNAs Related to Mouse Embryonic Brain Development from Available Transcriptomic Data

**DOI:** 10.1371/journal.pone.0071152

**Published:** 2013-08-14

**Authors:** Jie Lv, Wei Cui, Hongbo Liu, Hongjuan He, Youcheng Xiu, Jing Guo, Hui Liu, Qi Liu, Tiebo Zeng, Yan Chen, Yan Zhang, Qiong Wu

**Affiliations:** 1 School of Life Science and Technology, State Key Laboratory of Urban Water Resource and Environment, Harbin Institute of Technology, Harbin, China; 2 College of Bioinformatics Science and Technology, Harbin Medical University, Harbin, China; University of Torino, Italy

## Abstract

Long non-coding RNAs (lncRNAs) as a key group of non-coding RNAs have gained widely attention. Though lncRNAs have been functionally annotated and systematic explored in higher mammals, few are under systematical identification and annotation. Owing to the expression specificity, known lncRNAs expressed in embryonic brain tissues remain still limited. Considering a large number of lncRNAs are only transcribed in brain tissues, studies of lncRNAs in developmental brain are therefore of special interest. Here, publicly available RNA-sequencing (RNA-seq) data in embryonic brain are integrated to identify thousands of embryonic brain lncRNAs by a customized pipeline. A significant proportion of novel transcripts have not been annotated by available genomic resources. The putative embryonic brain lncRNAs are shorter in length, less spliced and show less conservation than known genes. The expression of putative lncRNAs is in one tenth on average of known coding genes, while comparable with known lncRNAs. From chromatin data, putative embryonic brain lncRNAs are associated with active chromatin marks, comparable with known lncRNAs. Embryonic brain expressed lncRNAs are also indicated to have expression though not evident in adult brain. Gene Ontology analysis of putative embryonic brain lncRNAs suggests that they are associated with brain development. The putative lncRNAs are shown to be related to possible cis-regulatory roles in imprinting even themselves are deemed to be imprinted lncRNAs. Re-analysis of one knockdown data suggests that four regulators are associated with lncRNAs. Taken together, the identification and systematic analysis of putative lncRNAs would provide novel insights into uncharacterized mouse non-coding regions and the relationships with mammalian embryonic brain development.

## Introduction

Recent transcriptomic researches have revealed that a significant fraction of genome can transcribe non-coding RNAs (ncRNAs), the proportion of which is much larger than previously anticipated [1], [2]. Over 90% of nucleotides in the human genome can be transcribed, as predicted by the ENCODE project [3]. In the ncRNA world, long non-coding RNAs (lncRNAs) which are manually defined by their size (at least 200 nt) are a distinct group from small RNAs (<200 nt, such as miRNAs and siRNAs). In general, lncRNAs are mRNA-like transcripts that lack long open reading frames and conserved secondary structures and show low sequence conservation, making it hard to be computationally identified from genome sequences [4]. It is speculated that lncRNAs can be transcribed by RNA PolII and are capped, spliced and polyadenylated (polyA) [5]. Previously considered to be ‘transcriptional noise’, lncRNAs are less expressed than protein-coding genes while in a highly tissue-specific expression pattern [6]. LncRNAs have diverse roles in genomic regulation, involving transcriptional regulation, imprinting and epigenetic regulation [7]–[9]. For instance, lncRNAs such as *Kcnq1ot1* and *Air* mediate the silencing of multiple genes in the *Kcnq1* and *Igf2r* imprinted gene clusters, respectively, by recruiting chromatin modifying machinery [10]–[13]. LncRNA *HOTAIR* expressed from *HOXC* cluster is shown to epigenetically repress the *HOXD* locus by involvement of the PRC2 complex [14]. In addition, Imprinted lncRNAs, such as *Rian* and *Mirg* [15]–[17], can also be precursors of small RNAs such as snoRNAs and miRNAs.

Embryonic brain development is a precisely and dynamically regulated process involving participation of many lncRNAs and also coding gene isoforms from alternative transcription and splicing [18]. A recent research reveals that genomic regions evolving more rapidly between human and other primates were located in several non-coding regions, including one brain-specific lncRNAs expressed in developmental stage of human cortex [19]. Other studies also suggest that lncRNAs might be involved in the development of brain [8], [20]–[22]. Though tiling arrays [23] are widely used to detect gene expression at different developmental stages, the information is limited for unannotated regions because they are unable to detect unknown gene structure [24]. RNA-sequencing (RNA-seq) allows sensitive identification of lowly expressed transcripts and is independent of current gene annotations [25], which is ideal for detecting novel transcripts, including lncRNAs [26]. RNA-seq has been used to identify thousands of long intergenic non-coding RNAs (lincRNAs) in human [6], [22], [27]–[29], mouse [24], [30], [31] and other species [32]–[36]. Several studies in mouse identified over 20,000 lncRNAs in various murine tissues and cell types by RNA-seq [24], [30], [31]. Mammalian embryonic brain development is a complex process involving synaptogenesis and cell differentiation. The characterization of embryonic brain related lncRNAs provides evidence regarding the roles of lncRNAs in brain function regulation [20], [37], [38]. For NONCODE lncRNAs, brain-specific lncRNAs account for 40% of the most differentially expressed 121 lncRNAs across 31 tissues/cell lines [29]. However, the current RNA-seq based strategy would miss expressed lncRNAs in narrow developmental time windows, especially in developmental periods. Systematic identification and characterization of organ development related lncRNAs are still few until now. Given the tissue-specific and developmental stage-specific expression patterns for lncRNAs, more lncRNAs would be identified when combining several independent transcriptomic data.

Here, we use publicly available RNA-seq data from embryonic brain tissues to excavate novel embryonic brain development related transcripts. Due to that many previous lncRNA studies focused on Embryonic Stem cells (ES cells) [24], [30], [39], we also analyze lncRNAs in ES cells to faithfully compare with embryonic brain lncRNAs. We build a pipeline to assemble, filter and report novel embryonic lncRNAs by unifying available RNA-seq data in developmental brain. After RNA-seq based de novo transcript identification and stringent filtering out of putative protein-coding potential transcripts, we obtain a confident set of 29,837 lncRNA transcripts in embryonic brain. Then, we characterize putative lncRNAs by diverse features including transcript structure, evolutionary conservation and chromatin data. A considerable number of putative lncRNAs are not supported by available annotations, such as Expressed Sequence Tags (ESTs), mRNAs and cross-species information. Gene Ontology (GO) enrichment analysis suggests that putative embryonic brain expressed intergenic and intronic lncRNAs are involved in brain development and transcription regulation, embryonic development and metabolic processes. It is suggested that the putative lncRNAs expressed in embryonic brain tend to be close to known imprinted genes. Collectively, the systematic characterization of embryonic brain expressed lncRNAs is expected to provide novel insights into the uncharacterized mouse genome regions and relationships with embryonic brain development.

## Results

### Transcriptome Reconstruction of Available Mouse Transcriptome Data in Embryonic Brain Reveals Novel Embryonic Brain lncRNAs

To systematically discover novel lncRNAs with potential regulatory functions in embryonic brain, we collected a set of 17 mRNA-seq datasets (Table S1) involving three stages that mark important developmental time points/stages: (1) Embryonic day 14.5 (E14) brain; (2) Embryonic day 15.5 (E15) brain and (3) Embryonic day 17.5 (E17) brain (refer to Materials and Methods). Furthermore, data from ES cell were also processed separately in same way to be used for comparison with putative embryonic brain lncRNAs. Though the initial aims of producing these publicly available data were mainly to analyze expression of known mRNAs and non-coding RNAs, it was viable to obtain known and novel mRNA-like lncRNAs based on the assumption that they are capped, spliced and polyadenylated. Generally, non-polyA+ RNAs are less stable than polyA+ RNAs [40] and non-polyA+ non-coding RNAs are not favorable for experimental studies. Though ribo-depleted RNA-seq can provide non-polyA+ non-coding RNA information, it is not meaningful to use different RNA data with different preparation methods. Therefore, we only analyzed datasets based on selection of polyA+ transcripts. On average, 84% of the initial reads could be aligned to the mm9 assembly of the mouse genome sequence. Aligned RNA-seq data were combined for same developmental time points/stages.

**Figure 1:**
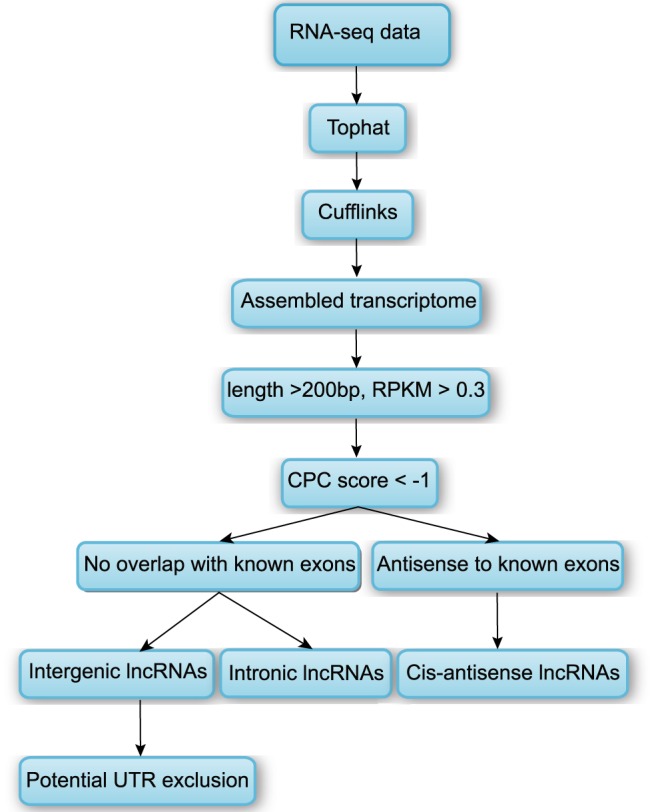
Pipeline for identification of lncRNA. Refer to main text for details.

As shown in Figure 1, the transcripts analyzed in this study were assembled using a widely used yet modified protocol [41]. Briefly, we used TopHat [42] to align reads of embryonic brain RNA-seq datasets. Then, we used Cufflinks [43] to assemble transcripts into known gene models or novel gene models by cufflinks guided by known gene annotations. The assembled transcripts were then merged by the Cuffmerge utility provided by the Cufflinks package, resulting in the assembly of 678,324 nonredundant transcript isoforms from 321,413 loci in embryonic brain, which provided the unique basis for further filtering putative transcripts and characterizing expression. These transcripts were concurrently annotated by Cuffcompare program in Cufflinks suite, of which known transcripts were used as reference to screen for novel lncRNAs. Small RNAs were filtered out using a minimum length threshold of 200 nt, further decreasing the number of transcripts to 421,379. In order to obtain a reliable dataset of putative lncRNAs, single exon genes were filtered out, unless supporting evidence from at least two developmental time points was available. Same procedure was also used in another study [44]. We also removed transcripts with Reads Per Kilobase per Million mapped reads (RPKM) <0.3 (refer to Materials and Methods). Applying the threshold, the number of transcripts in embryonic brain decreased to 72,544. Next, we removed transcripts that were likely to be assembly artifacts or PCR run-on fragments (refer to Materials and Methods). Among the different classes, only those annotated by “u”, “i”, “j” and “x” were retained, which represent novel intergenic, intronic, alternative spliced and cis-antisense transcripts, respectively. But here, most analysis focused on intergenic, intronic and cis-antisense lncRNAs.

The CPC program [45] was used to distinguish novel lncRNAs from protein-coding mRNAs, which was widely employed in lncRNA identifications [46]–[50]. We retained transcripts with CPC score<-1 (refer to Materials and Methods). However, CPC's SVM classifier could not accurately distinguish transcripts that fall entirely within UTR regions from those true non-coding transcripts. To deal with the limitation, we removed intergenic lncRNAs whose distances with nearest coding genes <1000 bp, resulting 29,837 lncRNA candidates involving embryonic brain, including 21,744 non-coding alternative spliced isoforms, 523 intergenic, 7488 intronic and 82 cis-antisense lncRNAs. Intronic as well as intergenic regions were also shown to be the major sources of non-coding RNAs [51]. Our results suggested that most of novel lncRNA candidates were regulated by alternative splicing, though not focused in this study. Intronic transcripts were another major sources of independent transcriptional units. In fact, previous studies revealed many alternative splicing patterns were associated with neurodevelopmental processes [52], [53].

Only 24 and 49 lncRNAs from the identified putative lncRNAs from embryonic brain and ES cell respectively overlapped with the intergenic lncRNAs identified by Guttman et al. [30], indicating embryonic brain lncRNAs were less likely to be expressed in ES cell, compared to putative ES cell lncRNAs. Of these putative embryonic brain lncRNAs, ∼82.1% had spliced EST support. The support rate was calculated as the proportion of lncRNAs with at least one nucleotide overlap with any previously annotated spliced ESTs (The calculation was also performed in following sections and were not stated again). Another 2.4% of embryonic brain lncRNAs were further supported by unspliced ESTs. The high EST coverage suggested that previously uncharacterized genomic loci may be an abundant source for studying lncRNAs.

**Figure 2:**
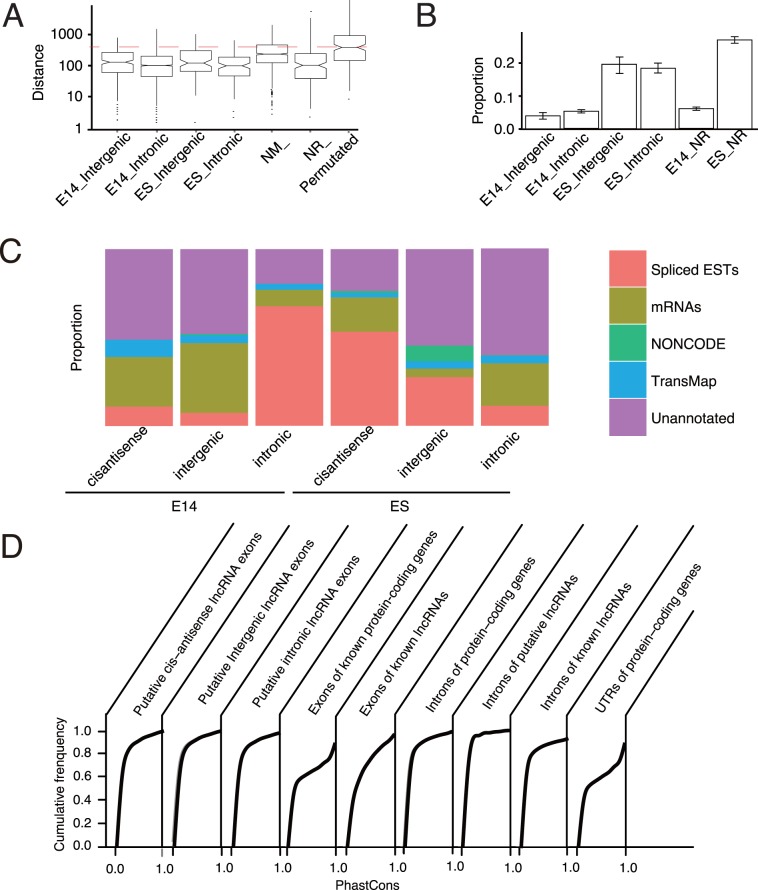
Genomic and transcriptional characterizations of putative embryonic brain lncRNAs. (A) Putative embryonic brain lncRNAs tend to have complete 5′ ends based on the annotation of CAGE clusters. The shortest distances of any CAGE clusters to all putative TSSs of putative lncRNAs are calculated. We compare the distances with that of putative ES lncRNAs, known genes and permutated putative embryonic brain lncRNAs. The red dash line indicates the median distance of permutated lncRNAs. (B) Few putative embryonic brain lncRNAs are translated. We apply Ribosome footprint data [57] to investigate the proportion of translation ability for our lncRNA sets. Remarkably here, we observe significant low proportion of putative lncRNAs in ES cell and embryonic brain, while comparable with known long non-coding genes (NR_) expressed in ES cell and embryonic brain. (C) Annotation of putative lncRNAs by genomic resources. Stacked plots are shown for putative lncRNAs mapped to spliced ESTs, known mRNAs, lncRNAs from NONCODE database, and the left are classified as “Unannotated”. To avoid redundant annotations among different genomic elements for lncRNAs, the order of annotation is forced bottom-up from ESTs to TransMap, leaving those without any annotations labeled as “Unannotated”. For putative embryonic brain lncRNAs, the major annotation sources are ESTs and mRNAs. (D) The cumulative distribution of median PhastCons scores across 33 placental mammal subset of species for putative embryonic brain lncRNAs, compared to introns of putative embryonic brain lncRNAs, known lncRNA and protein-coding genes, exons of known long protein-coding genes and known lncRNAs and UTRs of protein-coding genes. Only a small proportion of putative embryonic brain lncRNAs are highly conserved among mammals and most of those lncRNAs show low overall conservation level. Introns of putative embryonic brain lncRNAs, however, show even less conserved fractions, compared to putative lncRNA exons. X-axis, normalized PhastCons score; Y-axis, Cumulative frequency. NM_, known coding RefSeq genes; NR_, known long RefSeq genes; E14_, embryonic E14.5 brain; E14_NR, known lncRNA genes (NR_) expressed in embryonic brain; ES_NR, known lncRNA genes (NR_) expressed in ES cell; Permutated, positions of all lncRNAs are randomly chosen (details refer to Materials and Methods).

Novel lncRNAs may have a significantly shortened 5′ ends resulting from the 3′ bias in RNA-seq, which may be caused by polyA+ selection, fragmentation of cDNA and random hexamer priming [54]. To rule out of this, we characterized putative lncRNAs by CAGE clusters for the putative lncRNAs. Each CAGE cluster was enriched with various short CAGE reads which were termed as CAGE tags that were produced by high-throughput sequencing. We evaluated if putative Transcription Start Sites (TSSs) of putative embryonic brain lncRNAs tended to close to any CAGE clusters which were representative of potential TSSs. We calculated the shortest distance of any CAGE clusters to putative TSS from each putative lncRNA in embryonic brain. We also compared the distances with that of other lncRNA categories as well as permutated putative embryonic brain lncRNAs (refer to Materials and Methods). We observed that the putative embryonic brain lncRNAs were comparable with other lncRNAs, but distances are significantly lower than permutated embryonic lncRNAs (Mann-Whitney U test, *p*<2.0E-10, Figure 2A). The results suggested that the inherent 3′ bias of RNA-seq may not result in significantly shortened 5′ ends of putative lncRNAs by our pipeline.

It was still difficult to reliably distinguish non-coding RNAs from coding mRNAs or short peptides using only computational approaches [55]. Even, short ORFs can also be translated, therefore it is not reliable to judge translation of non-coding RNAs based on ORF length [56]. Recently, ribosome profiling, an approach based on sequencing of ribosome-protected RNA fragments, was carried out to screen for potential translation of mRNAs in mouse ES cell [57]. Surprisingly, a significant proportion of lncRNAs were predicted to be translated to short ORFs [57], [58], implying so-called ncRNAs can still produce short peptides. Therefore, analysis of lncRNA translation using experimental data was necessary. We applied the data from that study [57] to investigate the translation of our lncRNA sets. To make a reliable comparison of different types of lncRNAs, same number of known and putative ES cell lncRNAs with putative embryonic brain lncRNAs were sampled with replacement, while keeping comparable expression level. The sampling approach was also used in other analysis in next sections. Remarkably in Figure 2B, we observed significant low proportion (∼5%) of known and putative lncRNAs in embryonic brain. However, known and putative lncRNAs in ES cell tended more to be associated with ribosome footprints, consistent with the ES cell context from which ribosome profiling data were generated. A recent study estimated that about 92% of lncRNAs were not translated in two human cells [59], which further supported our results. Collectively, the evidence suggested that the majority of the putative lncRNAs may not be associated with translation machinery.

### Genomic and Transcriptional Characterization of Putative lncRNAs

For the putative embryonic brain lncRNAs, we then characterized their genomic and transcriptional features, compared to known protein-coding and known lncRNA genes expressed in embryonic brain when necessary. Firstly, as shown in Table 1, the length of putative embryonic brain intergenic lncRNAs was on average 605.2, comparable with those in previous studies [35]. The number of exons for putative embryonic brain intergenic lncRNA genes (on average, 1.32 exons) was less than known lncRNA genes (on average, 5.11 exons). Furthermore, the average exon number of putative embryonic brain intergenic lncRNAs was comparable with putative intergenic ES cell lncRNAs. As a more abundant group, 7488 putative embryonic brain intronic lncRNAs were more spliced with on average 1.6 exons and were comparable with putative ES cell lncRNAs. However, we may underestimate the exon number and length of putative lncRNAs, because their low expression levels may lead to incomplete assembly. We also successfully assembled 82 cis-antisense lncRNAs relative to known genes with ∼1.34 exons per transcripts and an average length over 1000 nt. The distributions of transcript length and number of exons for putative and known lncRNAs were shown in Figure S1 and S2. A full list of putative lncRNAs with details of genomic characterization was available in Table S2.

**Table 1:** Characterization of putative embryonic brain and ES cell lncRNAs with known lncRNAs for comparison.

	Intergenic embryonic brain	Intronic embryonic brain	Cis-antisense embryonic brain	Intergenic ES	Intronic ES	Cis-antisense ES	NR_ transcripts expressed in embryonic brain or ES cell
Exon number	1.32	1.6	1.34	1.21	1.42	1.29	3.42
Length	605.2	821.8	1423.4	1180.0	930.7	3728.9	2171.9
Number	523	7488	82	553	284	432	1461

When mapped to publicly available transcripts, it was indicated that 44.8% of putative intergenic lncRNAs in embryonic brain contained at least 10% overlap in exons with any annotations from Spliced ESTs, mRNAs, NONCODE lncRNAs [60] and the orthologous gene information in the TransMap annotation based on pairwise genome alignments from other vertebrate species (details of data refer to Materials and Methods). We found that 71.4% and 77.5% of putative intronic and cis-antisense lncRNAs in embryonic brain overlapped at least 10% in exon with any of these annotations (Figure 2C). For putative lncRNAs in embryonic brain, the major annotation sources were ESTs and mRNAs. Notably, NONCODE lncRNA annotations contributed only to putative intronic lncRNAs in ES cell, implying a possible bias towards ES cell in known lncRNA list (Figure 2C). Taken together, putative lncRNAs were poorly understood based on available genomic and evolutionary data.

Comparative genomic analysis of mouse lncRNAs indicated that their primary sequences, splice sites and promoters were under purifying selection [61]. However, the entire sequences may not be conserved, as lncRNA genes were significantly less conserved than protein-coding genes [6], [34]. To determine whether putative embryonic brain lncRNAs were evolutionary conserved, we investigated the average PhastCons scores across 33 placental mammal subset of species for putative embryonic brain lncRNAs (refer to Figure 2D). We found that only a small proportion of putative embryonic brain lncRNAs were highly conserved among mammals and most of putative embryonic brain lncRNAs contained conserved elements though showed low overall conservation level. Introns of putative embryonic brain lncRNAs, however in general, were even less conserved compared with putative lncRNA exons. The low overall conservation may result from rapidly evolvement of unnecessary lncRNA stretches, as many lncRNAs showed lineage specific conservation restricted to close species such as rat (data not shown).

According to a previous finding, ∼18% of TSS regions defined by CAGE was estimated to overlap repetitive elements [62]. We also found that ∼40% of known lncRNA genes (NR_) expressed in ES cell and embryonic brain overlapped with repetitive elements (>5% of length of lncRNAs), which were comparable with putative lncRNAs (Figure S3). The association of repeats for lncRNAs implied that the evolution of lncRNAs may be driven by repeat elements, which was also proposed by a recent study [63].

**Figure 3:**
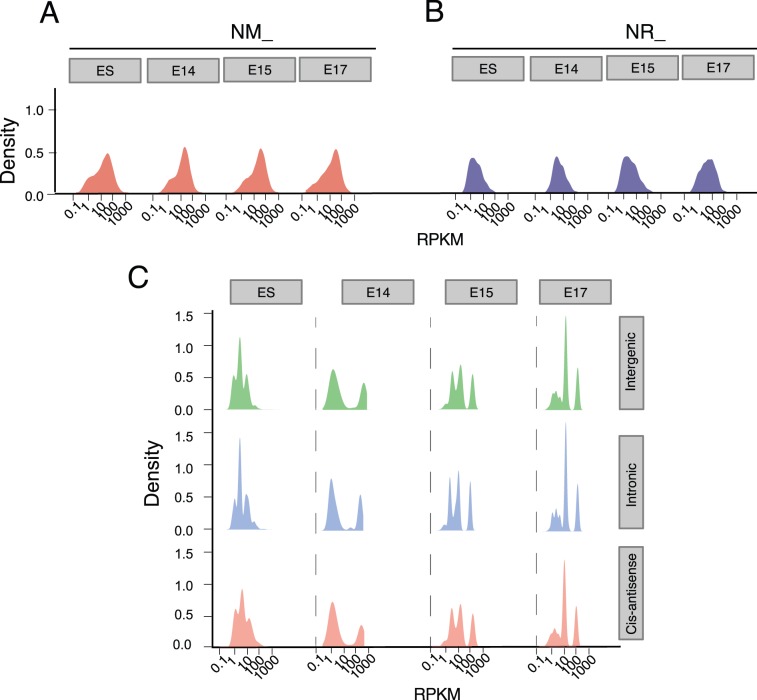
Expression distributions of known long protein-coding, lncRNA genes and putative embryonic brain lncRNA genes. The expression of (A) known long protein-coding (long NM_ RefSeq genes) and (B) known lncRNA genes (long NR_ RefSeq genes) and (C) putative embryonic brain lncRNAs over ES cell and developmental brain are compared. Consistent with previous studies, the expression of known long protein-coding RefSeq genes is significantly higher than known lncRNAs. The novel lncRNAs we identified are expressed at comparable levels with known NR_ transcripts. Notably, putative embryonic brain lncRNAs are expressed at about one tenth on average compared to known long protein-coding genes. Intergenic, intergenic lncRNAs; Intronic, intronic lncRNAs; Cis-antisense, cis-antisense lncRNAs. The gene expression (X-axis) is measured by RPKM which is a normalized metric for comparing gene expression of different genes.

Given the tissue and developmental stage expression specificity of lncRNAs, we then investigated the expression of putative and known lncRNAs expressed in different developmental stages. The expression of putative embryonic brain lncRNAs, known long protein-coding genes (NM_ RefSeq genes) and lncRNAs (NR_ RefSeq genes) over ES cell and developmental brain was shown in Figure 3A,B. Consistent with previous studies, the expression of known long protein-coding genes was significantly higher than known and putative embryonic brain lncRNAs. Previously untested transcriptional loci including our lncRNA sets would be expected to be expressed at lower levels. Indeed, the putative embryonic brain lncRNA genes were expressed at a low level, but comparable with known lncRNA genes (Figure 3C). Notably, putative embryonic brain lncRNAs were expressed at about one tenth on average compared with known protein-coding RefSeq genes. The lower lncRNA expression levels were consistent with previous reports [6], indicating it was a common property of lncRNAs. The overall expression increased gradually during brain development for known NR_ transcripts, but the trend was not evident for all types of putative embryonic brain lncRNAs.

Inspired by Liao et al.’s study [64], we further explored whether lncRNAs expressed in embryonic brain were restricted to embryonic stages. To this end, we compared expression patterns across different tissues for microarray probes (Mouse 430 2.0 array) overlapping putative embryonic brain lncRNAs (>99% genomic coverage for lncRNAs by probes) by BioGPS server [65]. Of the putative embryonic brain lncRNAs, we found that eight lncRNAs have matched probes for putative intergenic lncRNAs. Intronic and cis-antisense lncRNAs were not focused here because of potential ambiguous probe assignments. From Figure S4, we found that seven of eight probe-mapped lncRNAs were brain expressed and even brain-specific expressed, of which four lncRNAs were highly expressed markedly in brain. Therefore, putative intergenic lncRNAs in embryonic brain were also expressed in adult brain related organs and tissues. This phenomenon at least indicated that embryonic lncRNAs tended to continually express after mouse birth.

**Figure 4:**
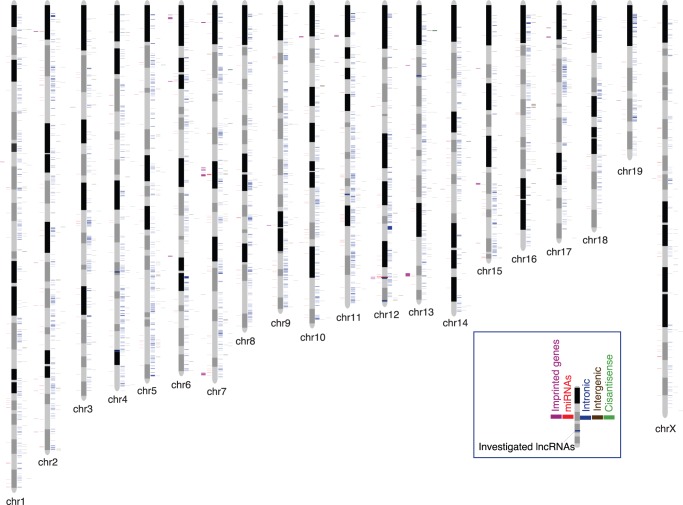
Chromosomal distribution of lncRNAs in the mouse genome. For each chromosome, the annotations shown from left to right are: Imprinted genes, miRNAs, intronic lncRNAs, intergenic lncRNAs and cis-antisense lncRNAs. For each chromosome, the chromosomal coordinates start from top (0) to bottom. Six lncRNA candidates investigated by RT-PCR with four developmental time points are marked by blue horizontal bars overlaid on chromosomes, while genomic details are in Table S3.

To further explore the lncRNA expression over brain development, we then randomly selected six putative lncRNAs specifically expressed in embryonic brain compared to ES cell and investigated their expression using RT–PCR for different developmental time points extending to 8-week whole brain (Figure S5, Figure 4). Conceptual translation of these lncRNAs revealed no obvious long ORFs (>100 amino acids) by ORF Finder (http://www.ncbi.nlm.nih.gov/gorf/gorf.html). Further details and annotations of our lncRNA locus models, together with whether these are validated by RT–PCR, were provided in Table S3. We found that except one lncRNA, others showed embryonic brain restricted expression patterns compared to 8-week whole brain. The false positive lncRNA had a low expression in RNA-seq data (RPKM <1.0), while the minimum of 1.0 RPKM was cited as required to obtain convincing expression in RNA-seq studies [66]. This result provided independent experimental evidence that most of embryonic brain specific lncRNAs were also, though lowly, expressed in adult brain, which implied that lncRNAs may play specific physiological roles in distinct developmental stages.

### Epigenomic Characterization of Putative lncRNAs

Though lncRNAs were considered as regulators of chromatin states, the transcription of lncRNA itself was also believed to be regulated by chromatin modifications [67]. A recent study characterized lncRNAs by DNA methylation and four histone modifications and found that lncRNA and protein-coding genes exhibited distinct chromatin patterns [67]. However, it was still unclear whether and how lncRNAs were regulated by specific chromatin marks and the degree of developmental stage specificity contributed by chromatin regulators, as the brain tissue showed a distinct chromatin pattern in Sati *et al*.’s study [67]. We analyzed ChIP-seq data of five representative chromatin marks in E14.5 brain for putative embryonic brain lncRNAs. To show the enrichment of chromatin modification signals in TSS proximal regions of the lncRNAs, we aligned ChIP-seq tags to the mouse genome (mm9) by Bowtie [68] and performed peak calling by MACS [41]. Enriched chromatin domains for five chromatin modifications were individually intersected with putative and known NR_ RefSeq lncRNAs by comparing genomic coordinates. Proportions of lncRNAs overlapped (at least 50% of lncRNA length) by chromatin marks were calculated as proportions of lncRNAs with any peaks of specific marks in TSS-proximal regions (5k upstream and 5k downstream of TSSs). We restricted this analysis to intergenic and intronic lncRNAs since unambiguous assignment of chromatin marks to the cis-antisense lncRNAs can be confounded by their exonic overlapping genes.

**Figure 5:**
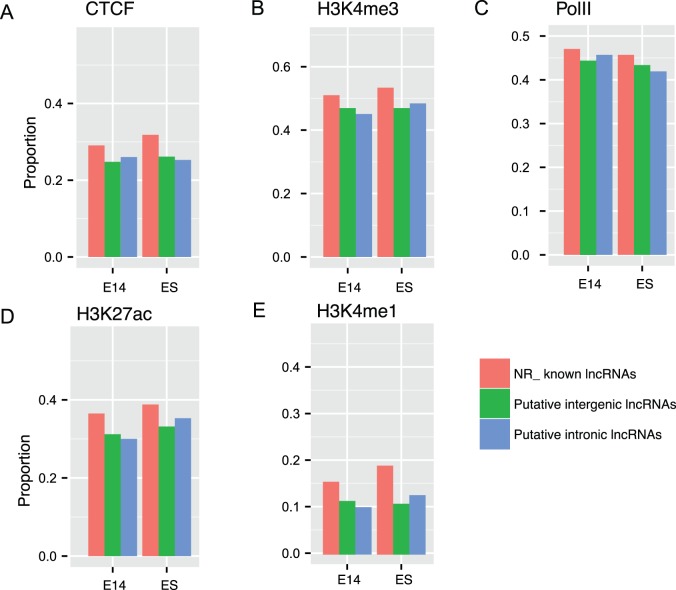
Proportions of lncRNAs that overlap with chromatin marks in TSS-proximal regions for all known long non-coding RNAs and putative lncRNAs, respectively. We analyzed ChIP-seq data of five representative chromatin marks for putative embryonic brain lncRNAs, that is (A) CTCF, (B) H3K4me3, (C) PolII, (D) H3K27ac and (E) H3K4me1. ChIP-seq tags are aligned to the mouse genome (mm9) by Bowtie and then are used to perform peak calling by MACS. Enriched chromatin domains for five chromatin modifications are intersected with putative and known lncRNAs by comparing genomic coordinates. Proportions of overlapped (at least 50% of lncRNA length) lncRNAs are defined by proportions of any peaks of specific marks in TSS-proximal regions (5k upstream and 5k downstream of TSSs) for known long non-coding RNAs and putative lncRNAs, respectively. We restrict this analysis to intergenic and intronic lncRNAs since unambiguous assignment of chromatin marks to the cis-antisense lncRNAs can be confounded by their exonic overlapping genes. NR_, known long RefSeq genes; E14_, embryonic E14.5 brain.

As expected, we found around a half of lncRNAs for putative intergenic and intronic lncRNAs in both ES cell and developmental brain were associated with H3K4me3 enriched domain, a hallmark indicative of transcription initiation of active genes in TSS-proximal regions, comparable with known lncRNAs expressed at same developmental stage (Figure 5B). Again, the result implied the identified 5′ ends of putative lncRNAs were close to bona fide TSSs. It was known that enhancer elements were characteristic of p300 with high H3K4me1 and H3K27ac and low H3K4me3 occupancy [69]. The lncRNAs with H3K27ac^+^H3K4me1^−^ signatures were once considered to be associated with active enhancers in Creyghton et al.’s study [70]. We were interested to investigate whether and to what extent the putative embryonic brain lncRNAs were enhancer related. As a result, we observed that lncRNAs were more associated with H3K27ac, which is more evident in known lncRNAs, compared to H3K4me1 (Figure 5D, E). Putative lncRNAs were also occupied by CTCF and PolII, which were comparable with known lncRNAs (Figure 5A, C). Therefore, the data indicated that ∼30% of putative lncRNAs were possibly enhancer related and around a half of putative lncRNAs were supported by active chromatin marks.

### Putative Embryonic Brain lncRNAs have Regulatory Roles in Brain Development

Nearly 40% of GENCODE v7 lncRNAs were estimated to flank protein-coding gene loci [29]. Intergenic lncRNAs were expected to have particular regulatory functions for nearby protein-coding genes. Recent studies also indicated that lncRNAs may affect gene expression of their neighboring genes in cis [6], [37], [71]–[74]. Manual inspection revealed many lncRNAs that were close to or within well-characterized protein-coding genes in embryogenesis, but showed distinct expression patterns. For example, we detected an intronic lncRNA (chr15∶66,090,924–66,092,050) that was selectively expressed in early embryonic brain and was then turned down to basal level in E17.5 brain, while the host gene *Kcnq3* was expressed increasingly in late fetal life to infancy [75]. *Kcnq3* was possibly important for regulating neuronal excitability, as shown by the *in situ* hybridization (ISH) data in the Allen Brain Atlas (ABA) website. As another example, we identified a lncRNA (chr14∶55,672,316–55,711,295) that was organized antisense to *Zfhx2*, which was different from the documented antisense transcript *zfhx2as* (chr14∶55,671,907–55,703,972) in lncRNAdb [76]. The *zfhx2as* transcript had the highest expression in E13–E16, but only had basal expression in brain after birth, while the novel antisense transcript peaked at E17 in our data (data not shown). The novel transcript we found in the loci suggested that there were also other transcript variants of *zfhx2as* for *Zfhx2* expression regulation in developing brain, consistent with available experimental evidence [77].

In vertebrates, enhancer elements can generate a class of transcripts termed eRNAs (enhancer RNAs), which were correlated with position adjacent protein-coding genes [71]. It was reasonable to explore relationships of putative intergenic lncRNAs and neighboring genes, given that lncRNAs were potentially enhancer related [78]. Closest known genes of putative embryonic brain lncRNAs were collected to detect enriched GO function terms [79], KEGG terms [80] and gene expression specificity terms UP_TISSUE (“Uniprot Tissue”) [81] using DAVID [82]. First, we investigated whether nearby genes of putative intergenic embryonic brain lncRNAs were enriched in any terms within the UP_TISSUE list, a curated list of gene expression specificity based on literature mining. Indeed, we found that genes expressed in brain and brain-related tissues in the list were significantly enriched by genes in the vicinity of putative intergenic embryonic brain lncRNAs as well as genes overlapping of intronic and cis-antisense lncRNAs, accounting for more than a half of them (Table S4). We then considered whether closest genes of putative intergenic lncRNAs were enriched in specific GO function terms, assuming that intergenic lncRNAs tended to regulate transcription of proximal genes rather than transcription of other genes. Our data suggested that these closest known protein-coding genes were enriched in GO categories involving brain development and transcription regulation. In addition, genes were also significantly associated with spliceosome assembly and ribonucleoprotein complex assembly, only detected by ChIPseeqer software [83] but not by DAVID, which was consistent with the scaffold function of lncRNAs [4].

Conceptually, transcription of intronic lncRNAs may interrupt the expression of their hosting genes. Previously, intronic lncRNAs were shown enriched in genes regarding transcription regulation and may have interaction with promoters to mediate hosting gene regulation [84]. Our data suggested that genes overlapping in intron with putative intronic lncRNAs in embryonic brain were enriched in GO categories related to a variety of biological processes including embryonic development, transcriptional regulation and metabolic processes. Metencephalon development term ranked first in the GO term list, followed by hindbrain development (Table S5). A KEGG term “mmu04360:Axon guidance” was significantly associated with putative intronic lncRNAs in embryonic brain (Table S6).

Cis-antisense lncRNAs were found to relate to gene regulation including alternative splicing and termination [85], genomic imprinting [86], X chromosome inactivation [87] and development [88]. Here, we analyzed the enriched GO categories of genes overlapping cis-antisense lncRNAs. However, no GO terms were enriched. A forebrain development term was insignificantly associated with cis-antisense lncRNAs. Though not statistically significant, this could be due to that the number of cis-antisense lncRNAs was relatively limited. Taken together, the GO and UP_TISSUE enrichments for each lncRNA type were generally in agreement with expectations, implying potential functions for putative lncRNAs in embryonic brain. The complete lists of the enriched UP_TISSUE, GO and KEGG terms and relevant information were shown in Table S4–S6.

### Putative Embryonic Brain lncRNAs are a Source of Potential Imprinted lncRNAs

**Figure 6:**
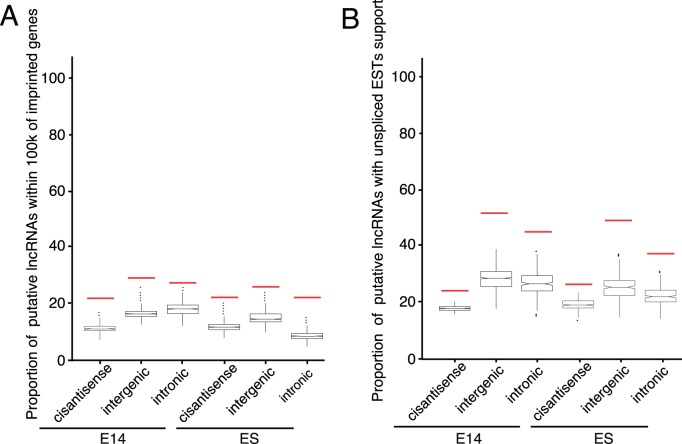
Putative embryonic brain lncRNAs are related to imprinted genes and unspliced ESTs. (A) Shown are the proportions of putative lncRNAs and permutated lncRNAs within 100k distance of compiled imprinted genes. Putative embryonic brain lncRNAs are involved in imprinted regions, comparable with putative lncRNAs in ES cell. (B) Shown are the proportions of putative lncRNAs and permutated lncRNAs that overlap with unspliced ESTs. Putative lncRNAs are significantly overlapped with unspliced ESTs. Embryonic, lncRNAs expressed at embryonic stages; ES, lncRNAs expressed at the ES stage; Red bar, the proportion of lncRNAs; Box, the proportion of permutated lncRNAs; whiskers denote the 10th and 90th percentiles.

Imprinting which was highly associated with antisense RNAs was shown to have important role in regulating brain development and function [2], [89]. Until now, lncRNAs were demonstrated to act in cis to induce the expression of other imprinted genes in imprinted clusters [90]. In addition, it was interesting to explore if any putative lncRNAs within imprinting clusters were potential imprinting lncRNAs. However, besides cis-antisense RNAs, whether lncRNAs of various types played a widespread functional role in imprinted cluster regulation was still uncertain. In addition, identification of imprinted lncRNAs from transcriptome data was viable, as a recent study demonstrated that imprinted lncRNAs can be identified by RNA-seq [91]. To this end, we examined whether putative lncRNAs in embryonic brain were close to or in known mouse imprinted clusters. We pre-compiled a list of imprinted transcripts, only kept 216 nonredundant entries with respect to genomic positions. We found that a significant proportion of putative intergenic and intronic lncRNAs expressed in embryonic brain were located within 100 kb of known imprinted clusters in which 43.1% of all imprinted transcripts were involved (empirical *p*-value <0.0005; Figure 6A). In ES cell, the tendency was comparable with that in embryonic brain. Taken together, putative embryonic brain lncRNAs may indeed relate to imprinted regulation.

Imprinted lncRNAs were usually unspliced, consistent with their nuclear localized property [92], in contrast to the non-nucleus localization of a majority of protein-coding genes. Here, putative embryonic brain lncRNAs were found to significantly overlap with unspliced ESTs (empirical *p*-value <0.0005, refer to Figure 6B). The putative lncRNAs in ES cell were also associated with unspliced ESTs in statistics. Altogether, it was implied that a large proportion of putative embryonic brain lncRNAs may excise regulatory roles in imprinting regions by transcription itself.

Then, we analyzed several lncRNAs in the well characterized *Dlk1*-*Dio3* imprinted cluster to investigate whether they themselves were potential imprinted lncRNAs. As an example, we found one putative embryonic brain lncRNA LncRNA_6 (Table S3) resided in the *Rian* locus exhibited an expression pattern comparable with that of the *Rian* locus [16], [17] during brain development from E12.5 to E18.5 brain. We also identified a putative intergenic lncRNA LncRNA_1 (Table S3) located in an imprinted cluster between *Rian* and *Mirg* flanked by mir882 and mir379. Given that mir379 was an imprinted miRNA [93], LncRNA_1, which was selectively expressed in embryonic E18.5 brain and was comparable in expression with transcripts in the *Rian* locus [16], [17], was a candidate imprinted lncRNA. Though not systematically investigated, our findings indicated that novel imprinted non-coding RNAs can be identified by our pipeline.

### Regulators Associated with Putative Embryonic Brain lncRNAs by Re-analyzing of RNA Interference Data

**Figure 7:**
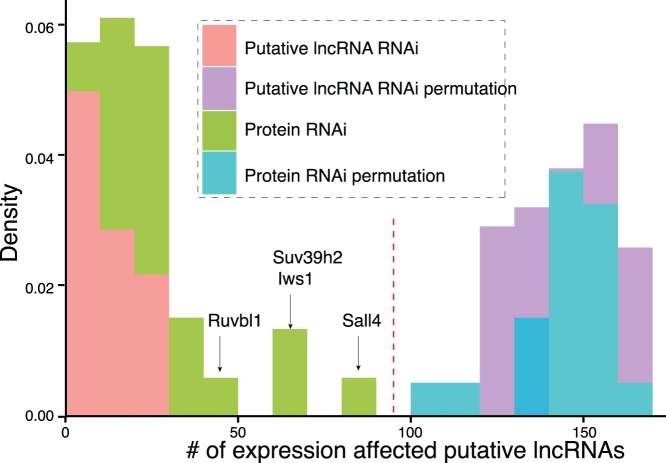
Distribution of putative embryonic brain lncRNAs and permutated lncRNAs with affected expression upon RNA interference of 226 lncRNAs and 40 regulatory proteins based on a published microarray data. Microarray probes aligned to putative embryonic brain lncRNAs by comparing genomic coordinates are used to explore the expression alteration of putative lncRNAs by comparing microarray profiles of RNA interference of 226 lncRNAs (and 40 regulatory proteins) and controls. The thresholds of 95th percentile and 5th percentile of ranked overall control expression in control profiles are used to obtain up- and down-regulated lncRNAs, respectively. Up-regulation and Down-regulation of putative lncRNAs are not separately analyzed for putative embryonic brain lncRNAs with altered expression. We observe that the distribution of putative embryonic brain lncRNAs with altered expression by RNA interference is separated from putative embryonic brain lncRNAs with altered expression by control interference by Red dash line.

Though at least thousands of lncRNAs were identified here and before, few were functionally characterized [10], [71], [73], [94], [95]. Previous studies demonstrated that intergenic lncRNAs were associated with transcription factors related to pluripotency [96] and it was estimated that ∼30% of lncRNAs can cooperate with specific chromatin regulatory factors to exercise trans-regulatory function [39]. We successfully aligned 187 from over 60,000 probes to putative embryonic brain lncRNAs in exon by comparing genomic coordinates (refer to Materials and Methods). We found that Knock Down (KD) of transcription factors or chromatin components affected expression of on average 25.5 putative embryonic brain lncRNAs (13.6 up-regulated versus 11.9 down-regulated) (Figure 7). Consistent with our expectation that KD of lncRNAs impacted little on other lncRNAs, we observed that KD of lncRNAs altered expression of down to on average ∼15.6 and maximum ∼28 putative embryonic brain lncRNAs, which can represent as negative controls (Figure 7).

We then tested whether putative embryonic brain lncRNAs were associated with chromatin components and transcription factors. We showed that Sall4, Suv39h2, Iws1 and Ruvbl1 were among the top four chromatin components with most altered expression after KD, while all of them were significantly different from permutated putative embryonic brain lncRNAs (Figure 7). Specifically, KD of Sall4, a known regulator of stem cell pluripotency, up-regulated 81 lncRNAs and down-regulated two lncRNAs. KD of Suv39h2, a histone H3K9 methyltransferase, was associated with aberrant expression of 67 lncRNAs, including three up-regulated and 64 down-regulated lncRNAs, implying potential epigenetic regulation involving lncRNAs. Last, Iws1 and Ruvbl1 were two nearly untouched proteins in literature and had no known links with lncRNAs in mouse until now. Here, KD of Iws1 was associated with the up-regulation of 62 lncRNAs and down-regulation of two lncRNAs. KD of Ruvbl1 was only associated with the down-regulation of 47 lncRNAs. We presented that a large number of lncRNAs were potential regulators for embryonic brain development. Taken together, the data pointed out the existence of potential functional lncRNAs which were associated with specific key transcription factors and chromatin components in transcriptional regulation.

## Discussion

In this paper, publicly available RNA-seq data from embryonic brain tissues were collected and mined to excavate novel embryonic development related transcripts. To this end, we have built a pipeline to assemble, filter and report novel embryonic brain lncRNAs. After RNA-seq-based de novo transcript identification and stringent filtering out of putative protein-coding potential transcripts, a confident set of lncRNA transcripts is obtained. We then characterize putative lncRNAs by diverse features including transcript structure, evolutionary conservation, chromatin information, known transcript annotations, CAGE, ribosome profiling data and RNA interference data. Collectively, the systematic characterizations of embryonic brain expressed lncRNAs are expected to provide novel insights into the uncharacterized mouse genome regions and the relationships with mammalian embryonic brain development.

Earlier, the functional significance of lncRNAs is quite controversial [61], [84]. The most of mammalian genome were considered to have nearly no functions and most of non-coding transcripts might be experimental artifacts [97]. However, a large number of tissue-specific and development-specific lncRNAs and related function analysis argue against that they are simply transcriptional noise [14], [71], [94], [98], [99]. More and more evidence indicates that genome is pervasively and specifically transcribed [3], [62]. Consistent with these, our studies revealed many more lncRNAs that were not reported in previous studies, even many were not annotated by relatively comprehensive genomic annotation data. Our data imply that a large number of lncRNAs are expressed in embryonic brain. It can be expected that when expanding the research targets, such as more normal state and perturbated tissues/cells, more lncRNAs can be identified [74], [98], [100]. When expanding the surveyed tissue types, we analyze the expression of eight intergenic lncRNA matched probes in Mouse 430 2.0 array in other tissues/cell types. Unexpectedly, most of these probes were specifically expressed in brain tissues, at least suggesting the putative lncRNAs may have continuous expression pattern after birth.

Several lncRNAs have recently been found to be associated with enhancers [71]. A plausible mechanism is that lncRNAs can act through transcription factors to regulate local chromatin remodeling, which in turn might enhance nearby gene activation [101]. Our data indicate that <40% of embryonic brain lncRNAs are associated with potential enhancer mark H3K4me1 and <10% for H3K27ac mark, respectively (Figure 5). In addition, ∼40% of putative lncRNAs both in embryonic brain and ES cell are associated with H3K4me3, a representative mark indicative of active transcription initiation. Several studies have indicated that lncRNAs can be independently transcribed and be positively correlated with neighboring coding genes [37], [72]. Jia et al. suggested that most of lncRNAs near protein-coding genes had chromatin marks related to independent mRNA transcripts [48]. Based on the above studies, it is reasonable to assume most putative long non-coding transcripts filtered by our pipeline are independent of neighboring genes and enhancer.

Mapping of the RNA-seq data from embryonic brain reveals a significant number of intronic lncRNAs, constituting the major component of the putative lncRNA repertoire. Recently, Klevebring et al. reported that ∼50% of the intronic transcripts were transcribed from antisense strand [102], contrary to respective hosting gene transcription. Therefore, the intronic lncRNAs detected here may have antisense characteristics, although untested because single-ended RNA-seq data lacked strand information. GO enrichment of genes that are overlapped with putative intronic lncRNAs further indicates that the putative embryonic brain intronic lncRNAs are closely related to biological processes including embryonic development, transcriptional regulation and metabolic processes. Altogether, intronic lncRNAs may play important roles in regulating gene expression during embryonic brain development.

The putative lncRNAs are comparable in length and exon number with that in other studies, but significantly less than known lncRNAs, which could be caused by low expression levels that could lead to incomplete assembly. Moreover, a large number of lncRNAs in embryonic brain are unspliced (empirical *p*-value <0.0005), which may imply that many lncRNAs are functional in nucleus (refer to Figure 6B). A recent work suggested that the transcription of *Airn*, spliced or unspliced, can suppress *Igf2r* promoter, which is more important than *Airn* transcription product [13]. A recent study also suggested that the transcriptional process of cis-antisense lncRNAs rather than transcriptional product regulated the overlapping imprinted protein-coding genes in the *Gnas* cluster [103]. Taken together, these examples at least imply that many characteristics of lncRNAs, such as lack of conservation, shorter transcripts [62], few exons and large variation in lncRNA stability [40], are consistent with inefficient splicing of lncRNAs. However, whether the unspliced tendency of lncRNAs is caused by relatively not deep sequencing is not easy to answer though many RNA-seq data are integrated, which would be efficiently explained by the availability of more RNA-seq data and the continued development of sequencing technology.

Furthermore, KD of lncRNAs often leads to significant transcriptional perturbation [39]. Our data further show that KD of transcription factors or chromatin components can lead to expression changes of average ∼25 putative embryonic brain lncRNAs, significantly less than controls, suggesting widespread yet specific regulatory functions for lncRNAs. More characterization of lncRNAs by loss of function experiments would be helpful to thoroughly elucidate regulatory function of putative lncRNAs.

Previously, most studies focused on lincRNAs, as the surrounding transcript structure of lincRNAs is simpler than non-coding RNAs overlapping genic regions. Other merits include the decrease of potential genomic noises and the exclusion of other transcripts in following experimental validation. The number of our putative intergenic lncRNAs is comparable with that of Guttman et al. [30], but few of which overlap with those reported in ES cells in their study, which is not unexpected, given that the tissue specific nature of lncRNA expression and different identification pipelines. Although RNA-seq provides information useful for non-coding RNA identification, it would be necessary to further explore the lncRNA world by integrating different technology, such as ChIP-seq, and poly(A)-site seq, though beyond the scope of this study. We envision that combination of different data sources including sequencing data and functional genomics data by more effective high-throughput data processing algorithms and pipelines would greatly enhance the understandings of lncRNA function and help identify additional lncRNAs for functional studies.

### Conclusions

Based on the publicly available mRNA-seq data from embryonic brain, we excavate putative embryonic brain development related lncRNAs based on a customed pipeline. We characterize putative lncRNAs by expression, genomic annotation and epigenomic data, confirming the validity of our customed pipeline. The putative embryonic brain lncRNAs show significant association with neighboring genes having regulatory function in brain development and transcription regulation. Furthermore, putative embryonic brain lncRNAs tend to close to or themselves are potential imprinted genes. Chromatin regulators Sall4, Suv39h2, Iws1 and Ruvbl1 are most likely to associate with putative embryonic brain lncRNAs. Taken together, the systematic analysis of putative lncRNAs would provide novel insights into uncharacterized mouse genome regions and into the relationships with embryonic brain development.

## Materials and Methods

### Ethics Statement

Care and handling of all experimental animals used in this work were conducted in accordance with Harbin Institute of Technology’s institutional animal care and use committee policies and all efforts were made to minimize suffering. The protocol was approved by School of Life Science and Technology, State Key Laboratory of Urban Water Resource and Environment, Harbin Institute of Technology (Permit Number: 2012–56).

### Datasets

Seventeen PolyA+ RNA-seq datasets from different stages of developmental brain tissues and six datasets from ES cells were collected from the ENCODE project [104] and were downloaded from the NCBI SRA website. The sample information of RNA-seq data are listed in Table S1.

Known long coding and lncRNA gene annotations were compiled from RefSeq [105] and Ensembl [106] gene annotation with further filtering of length >200 nucleotides.

Other data used for lncRNA analysis including small RNA-seq and ChIP-seq data were also downloaded from the NCBI SRA website [107]. Known gene information and annotation data (ESTs, mRNAs and Transmap) used for lncRNA annotations were downloaded from UCSC [108] and another known lncRNA list was downloaded from the NONCODE database [60]. Known imprinted genes were manually compiled based on a compiled list from NCBI GEO with the accession number GSE27016 [109]. The CAGE cluster data were downloaded from the FANTOM4 website [110]. The processed Ribosome footprint profiling data [57] were downloaded from NCBI GEO with the accession number GSE30839 (only processed files of GSM765295 and GSM765298 were used). Chromatin modification ChIP-seq data of ES and E14 brain were download from UCSC with the accession number GSE31039. Expression microarray data of knockdown of lncRNAs and proteins were downloaded from NCBI GEO with the accession number GSE30245.

### RNA-seq Data Analysis

Sequencing reads in FASTQ format were mapped to mouse genome (mm9) and novel splice junctions were automatically determined using TopHat (version 1.4.1) [42], with default parameters except “-G” option together with Gene Transfer Format (GTF) file of Ensembl gene annotation [106] used for read mapping, followed by a rRNA removal step based on collected rRNA sequences from NCBI [105]. Unmapped reads of RNA-seq data from mouse strain different from C57BL/6 were aligned to SNP corrected mouse genome sequence according to the data processing illustration at GEO (GSE22131). Splice junctions after initial de novo mapping were compiled and merged with splice junctions of known genes in initial genome mapping, which were used in the final round of mapping. The pool of mapped reads were merged separately for each stage of E14.5, E15.5 and E17.5 brain. Only reads with Phred score >20 were kept. Because the amounts of data varied from different sources, an equal number of reads were sampled without replacement based on the stage with the minimum read number. The sampled alignment data were then fed to an assembler Cufflinks (version 0.9.3) to assemble aligned reads into transcripts [43]. The data of ES cell were also processed following a similar protocol.

Transcript abundances were estimated by Cufflinks in Fragments Per Kilobase per Million mapped reads (FPKM) for paired-end reads or Reads Per Kilobase per Million mapped reads (RPKM) for single-end reads [66]. All transcripts identified by Cufflinks were matched to and guided by the RefSeq and the Ensembl gene models (excluding RefSeq overlap) by Cuffcompare which was included in the Cufflinks suite. To effectively address the issue that a sequenced read can align to different isoforms of the same gene, Cufflinks used maximum likelihood estimation based on a numerical optimization algorithm. By using customed scripts, the resulting files were further analyzed to extract candidate lncRNAs as well as known coding and non-coding genes.

### Filtering of Putative lncRNAs Transcripts

We filtered the assembled novel transcripts from different developmental stages of brain as well as novel transcripts from ES cell to obtain putative lncRNAs. Firstly, identical and overlapping transcripts were merged to remove redundancy. Then, transcripts overlapping with known exons of genes were removed. Only transcripts with length >200nt were retained. In order to obtain a reliable dataset of putative lncRNAs, single exon models were filtered out unless supporting evidence from at least two developmental time points was available. Next, we removed transcripts that were likely to be assembly artifacts or PCR run-on fragments according to class code annotated by Cuffcompare. Among the different classes, only those annotated by “u”, “i”, “j” and “x” were retained, which represent novel intergenic, intronic, alternative spliced and cis-antisense transcripts, respectively. But here, most analyses were focused on intergenic, intronic and cis-antisense lncRNAs. Extremely low gene expression is generally considered to be transcriptional noise [111]. On average, 84% of the initial reads could be aligned to the mm9 assembly of the mouse genome sequence. Transcripts with RPKM/FPKM under lower bound of single tail 84% confidence interval (<0.3) for all expression values were removed. The bottom 16% expression signals were considered as noises, which was deduced from the average mapping rate in RNA-seq read alignment. Interestingly, the threshold of 0.3 was also used by a systematic transcriptomic study to balance the false negatives and false positives [112], justifying the suitability of threshold determination.

Lastly, we calculated the protein-coding capacity of novel transcripts using CPC which incorporates the sequence features into a support vector machine to assess the protein-coding potential of each transcript. CPC used six features extracted from nucleotide sequences to define transcripts as coding if conceptual translations were long and were similar to known proteins. The proportion of coding transcripts miss-classified as non-coding RNAs by CPC was previously shown to be marginal [34], [35], suggesting CPC is a robust approach for distinguishing coding from noncoding RNAs. Then those putative transcripts with CPC score<−1 were retained as candidate lncRNAs for the further analysis. However, CPC’s SVM classifier could not accurately distinguish transcripts that fall entirely within UTR regions from those true non-coding transcripts. In most mammalian genomes, the 3′ UTR regions of a coding transcript may extend for several kilobases (kb) and were abundant in many EST libraries. To explore influences of the limitation, we manually searched dozens of intergenic lncRNAs proximal to coding gene against UTRs from UTRdb [113] but found no overlap with UTRs. To eliminate the potential inclusion of unannotated UTRs (Untranslated Regions) and also promoter related divergent transcripts, we removed intergenic lncRNAs whose distances with nearest coding genes <1000 bp.

Other than putative lncRNAs in embryonic brain and ES cell, we also quantified the expression of known lncRNAs. To make a reliable comparison with putative embryonic brain lncRNAs, same number of known lncRNAs with putative embryonic brain lncRNAs were sampled with replacement, while keeping comparable expression level.

### Classification of Putative lncRNAs

The assembled putative lncRNAs were divided into three categories: (1) lncRNAs without any overlaps with any genes (RefSeq or Ensembl) were classified as intergenic overlap lncRNAs (intergenic lncRNAs); (2) lncRNAs that were entirely contained within intron of any protein-coding genes in either sense or antisense orientation were classified as intronic overlap lncRNAs (intronic lncRNAs); (3) lncRNAs with exonic overlaps with any exons of RefSeq transcript on the opposite strand were classified as cis-antisense overlap lncRNAs (cis-antisense lncRNAs).

### Permutation Tests

Permutated lncRNAs were generated based on putative lncRNAs in embryonic brain to control for putative lncRNAs by bedtools (http://code.google.com/p/bedtools/). We avoided sampling repeat masked regions and RefSeq gene regions that were download from UCSC by a particular parameter -excl in bedtools for putative intergenic lncRNAs while keeping comparable length with putative intergenic lncRNAs. Only introns of Ensembl genes were sampled to obtain permutated lncRNAs for putative intronic lncRNAs. We also avoided sampling repeat masked regions with parameter -excl in bedtools for putative cis-antisense lncRNAs. 10,000 permuations were performed for each analysis, in which false positive rate <0.05% was considered as statistically significant.

### Conservation Analysis

PhastConsElements30way data for mouse genome (mm9) downloaded from the UCSC database were used to investigate conservation of lncRNAs. To assign a conservation score to a transcript, the average PhastCons score for the concatenated exonic regions of each transcript model was calculated. The conservation score of putative embryonic brain lncRNAs (putative intronic, intergenic and cis-antisense lncRNAs, separately) was compared with introns and UTRs of known long protein-coding genes and known lncRNA genes as well as exons of known genes (known long protein-coding genes and lncRNA genes).

### Gene Function Enrichment Analysis by DAVID

DAVID [82] was used to perform gene function enrichment analysis based on GO [79], KEGG [80] and UP_TISSUE [81] annotation by submitting closest gene lists for putative embryonic brain intergenic lncRNAs, host genes for putative embryonic brain intronic lncRNAs and overlapping genes for putative embryonic brain cis-antisense lncRNAs, respectively. Only putative embryonic brain intergenic lncRNAs with distance to closest genes <500 kb were kept for this analysis, which would eliminate long-distance irrelevant genes. Furthermore, putative embryonic brain intronic lncRNAs embedded in long introns (>100 kb) of known genes were also discarded to avoid the bias of large introns. Functional terms with Benjamini-Hochberg adjusted *p*-values <0.05 were considered to be significantly enriched.

### Mouse Tissue Preparation, RNA Preparation and RT-PCR

C57BL/6 mice were time mated overnight. Total RNAs were extracted from the brain tissue at E12, E15, E18 and 8 week by Trizol reagent (Invitrogen, Eugene, USA). Noon on the day of the presence of a plug was considered to be embryonic day 0.5 (E0.5). E0.5 was omitted to simplify the notation by default throughout the paper.

Agarose gel electrophoreisis was used to identify total RNA integrity. The ratio of A260:A280 was used to indicate the purity of total RNAs. The cDNAs were synthesized using a SuperScriptTMIII RNase H-Reverse Transcriptase kit (Invitrogen, Eugene, USA). Subsequently, cDNAs were used for genes expression analysis of lncRNA candidates by semi-quantitative RT-PCR.

Semi-quantitative RT-PCR was performed using Taq DNA polymerase (TaKaRa, Dalian, China), and the reaction was performed 30 cycles. The β-actin (Accession No. NM_007393, nt. 520–717, sense: 5′-taccacaggcattgtgtaggact-3′, antisense: 5′-ttgatgtcacgcacgatttccct-3′) was also performed 30 cycles and used as a loading control. A DNAse treatment step was performed to remove possible genomic DNA contamination, which was further confirmed by testing a known intron genes (not shown). The details of experimentally investigated lncRNAs were listed in Table S3. All PCR products were of the expected sizes, as shown by gel electrophoresis. All primer sequences were designed not to amplify nonspecific sequences and they did not target repeat elements (Table S7).

### Processing of the RNA Interference Microarray Data

We aligned the probes of Agilent Mouse 60 K lincRNA Array customized by Guttman et al. [39] to exons of our putative embryonic brain lncRNAs to investigate if any probes can represent our putative lncRNAs and quantify the expression levels. Although the lncRNAs in Agilent Mouse 60 K lincRNA array were used to study ES pluripotency, 187 from over 60,000 probes can be aligned to putative embryonic brain lncRNAs in exons by comparing genomic coordinates (>99% overlap). Based on the customized array, Guttman et al. chose 40 proteins and 226 intergenic lncRNAs to knock down and screened the genome-wide expression. They were knocked down and were measured the expression profiles with two replicates for each, accounting for 532 microarray profiles altogether. Fifty-five non-experiment microarray profiles were used to control for RNA interference. The thresholds of 95th percentile and 5th percentile of ranked overall control expression in control profiles were used to obtain up- and down-regulated lncRNAs, respectively.

## Supporting Information

Figure S1 **The distribution of transcript length for putative embryonic brain and ES cell lncRNAs and known NR_ lncRNAs.** (A) The distribution of transcript length for putative embryonic brain and ES cell lncRNAs. (B) The distribution of transcript length for known NR_ lncRNAs expressed in embryonic and ES cell, respectively.
(TIF)

Figure S2 **The distribution of exon number for putative embryonic brain and ES cell lncRNAs and known NR_ lncRNAs.** (A) The distribution of exon number for putative embryonic brain and ES cell lncRNAs. (B) The distribution of exon number for known NR_ lncRNAs expressed in embryonic and ES cell, respectively.
(TIF)

Figure S3 **The proportion of putative lncRNAs and known lncRNAs that overlap with repeat elements.** About 40% of putative and known lncRNAs are associated with repeat elements (>5% of length of lncRNAs). NR_, known non-coding RefSeq genes; E14, embryonic E14.5 brain.
(TIFF)

Figure S4 **The distribution of expression across adult tissues (Mouse 430 2.0 array) for probes matched with putative embryonic brain lncRNAs. The expression information is taken from** BioGPS server [65]. We obtain eight lncRNAs which are associated with non-redundant probes, of which seven probes are brain expressed and even brain-specific expressed. Even, four probes are highly expressed markedly in brain. Therefore, putative intergenic lncRNAs in embryonic brain are also expressed in adult brain related organs and tissues.
(JPG)

Figure S5 **Chromosomal distribution of lncRNAs in the mouse genome and randomly chosen putative embryonic brain lncRNAs for exploring expression in adult whole brain.** For each chromosome, the chromosomal coordinates start from top (0) to bottom. Six lncRNA candidates are marked by blue horizontal bars overlaid on chromosomes and are investigated by RT-PCR with four developmental time points (refer to Materials and Methods), while genomic details are in Table S3. LncRNA_x represents putative embryonic lncRNA ID in Table S3.
(TIF)

Table S1 **RNA-seq genome mapping using Tophat.**
(XLS)

Table S2 **Details of putative embryonic brain lncRNAs by RT-PCR.**
(XLS)

Table S3 **Full list of putative embryonic brain lncRNAs.**
(XLS)

Table S4 **Gene function enrichment analysis based on UP_TISSUE annotation for putative embryonic brain lncRNAs.**
(XLS)

Table S5 **Gene function enrichment analysis based on Gene Ontology (GO) annotation putative embryonic brain lncRNAs.**
(XLS)

Table S6 **Gene function enrichment analysis based on KEGG annotation putative embryonic brain lncRNAs.**
(XLS)

Table S7 **Primer designs for six putative embryonic brain lncRNAs.**
(XLS)
