# Supplementary figures and images for: Identification and Characterization of Long Non-Coding RNAs Related to Mouse Embryonic Brain Development from Available Transcriptomic Data
Source: PLoS One. 2013 Aug 14;8(8):e71152. doi: 10.1371/journal.pone.0071152 (PMC3743905; doi:10.1371/journal.pone.0071152)

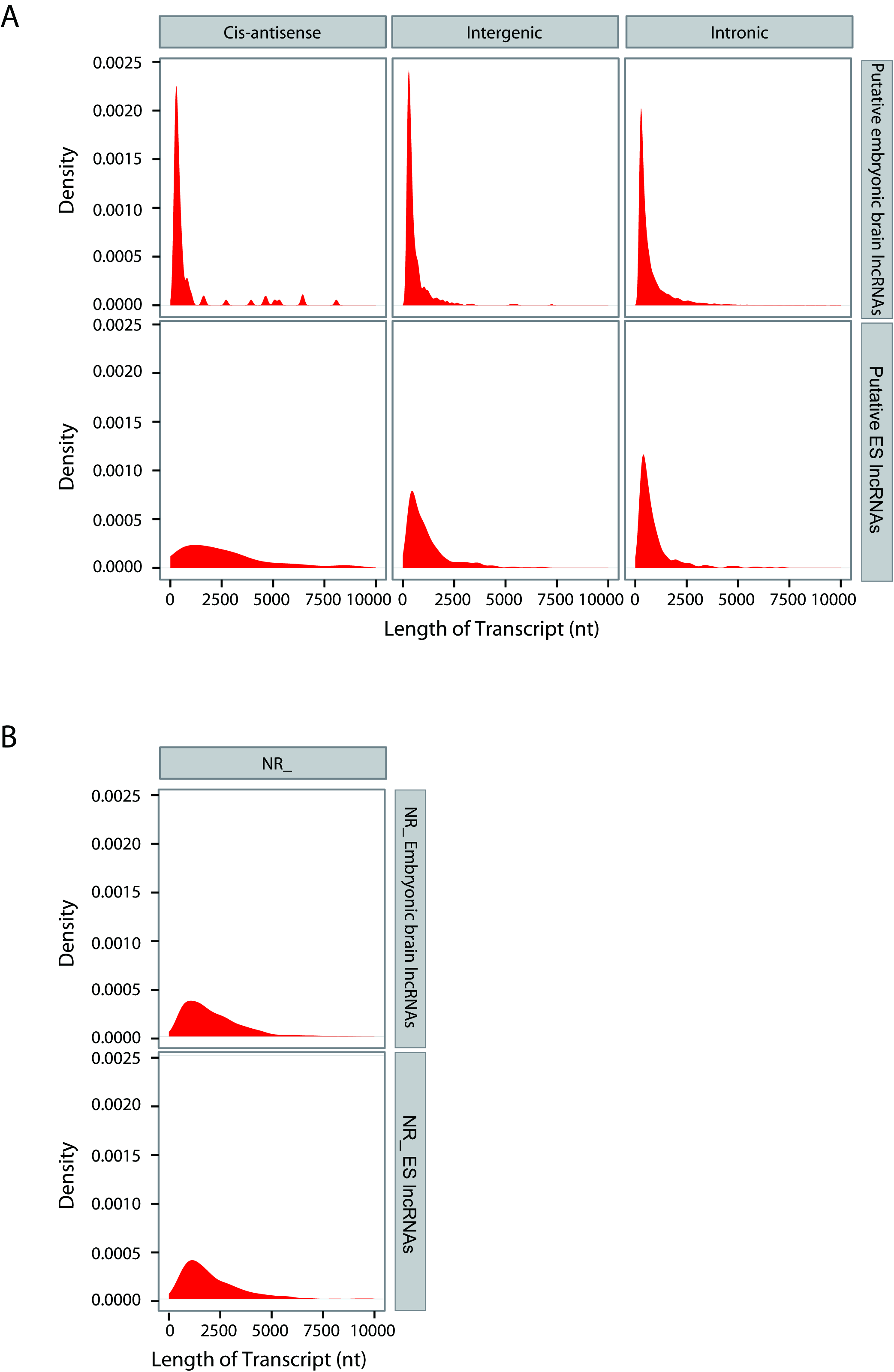

Supplement: Figure S1 — The distribution of transcript length for putative embryonic brain and ES cell lncRNAs and known NR_ lncRNAs. (A) The distribution of transcript length for putative embryonic brain and ES cell lncRNAs. (B) The distribution of transcript length for known NR_ lncRNAs expressed in embryonic and ES cell, respectively. (TIF) [file pone.0071152.s001.tif]

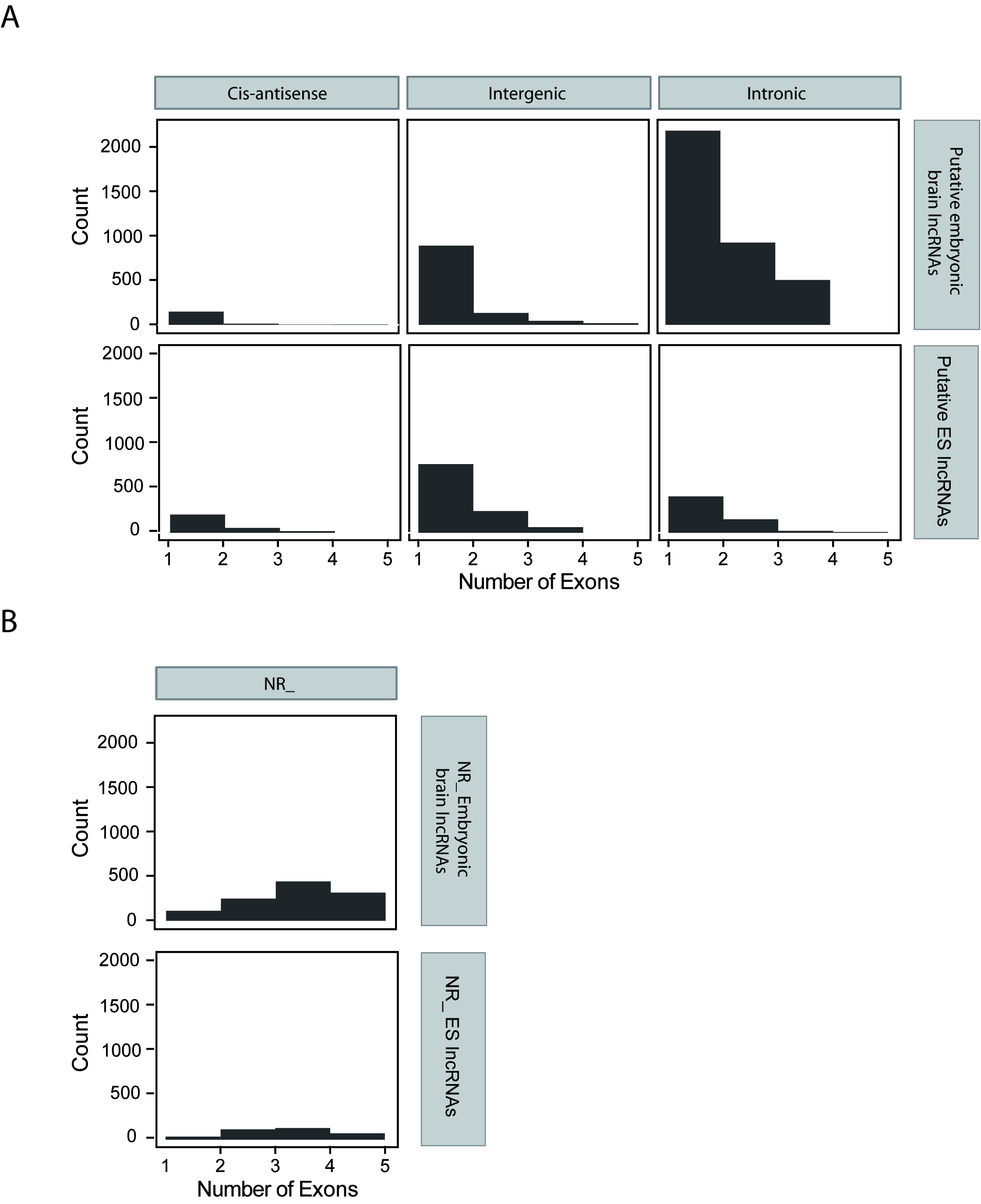

Supplement: Figure S2 — The distribution of exon number for putative embryonic brain and ES cell lncRNAs and known NR_ lncRNAs. (A) The distribution of exon number for putative embryonic brain and ES cell lncRNAs. (B) The distribution of exon number for known NR_ lncRNAs expressed in embryonic and ES cell, respectively. (TIF) [file pone.0071152.s002.tif]

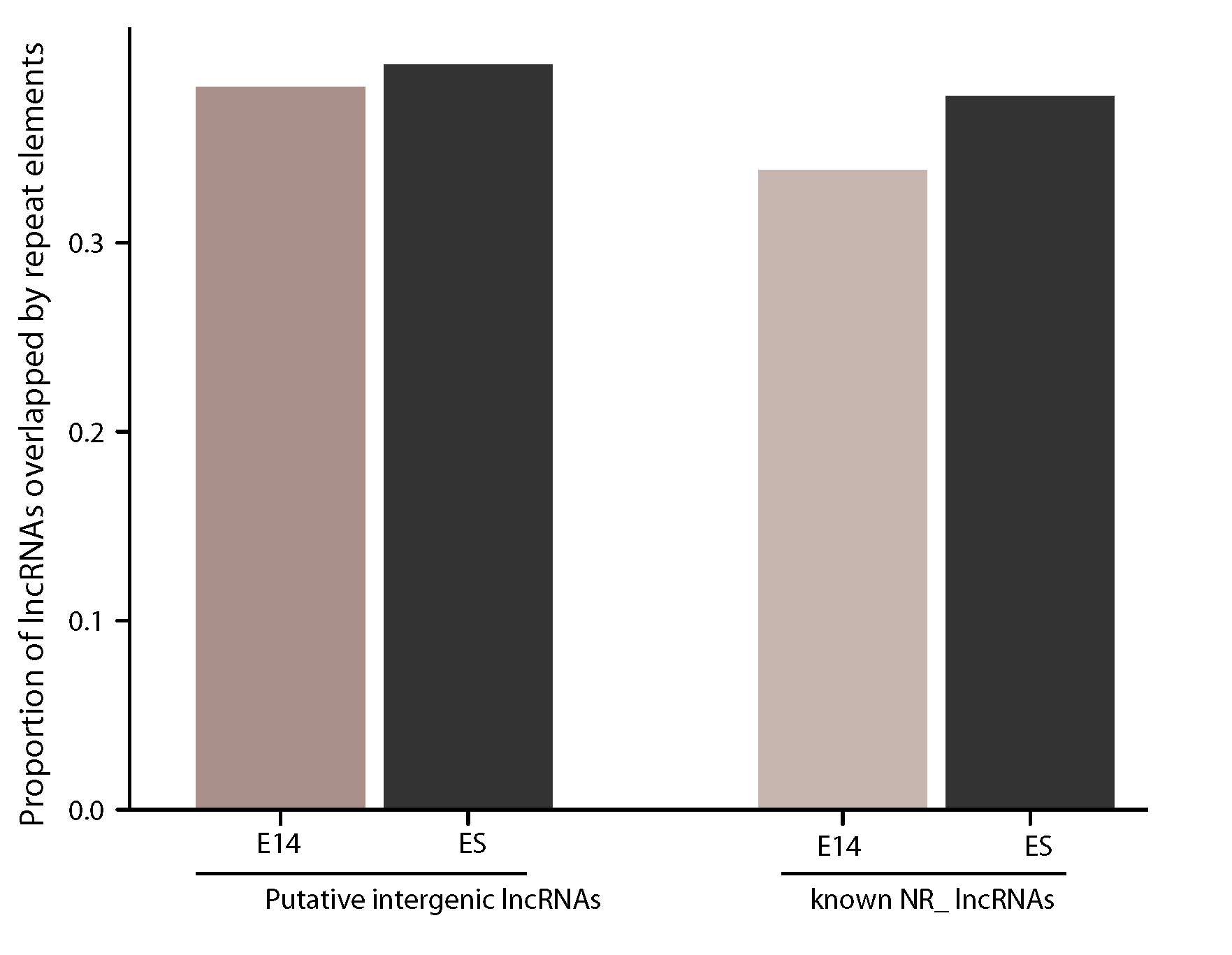

Supplement: Figure S3 — The proportion of putative lncRNAs and known lncRNAs that overlap with repeat elements. About 40% of putative and known lncRNAs are associated with repeat elements (>5% of length of lncRNAs). NR_, known non-coding RefSeq genes; E14, embryonic E14.5 brain. (TIFF) [file pone.0071152.s003.tiff]

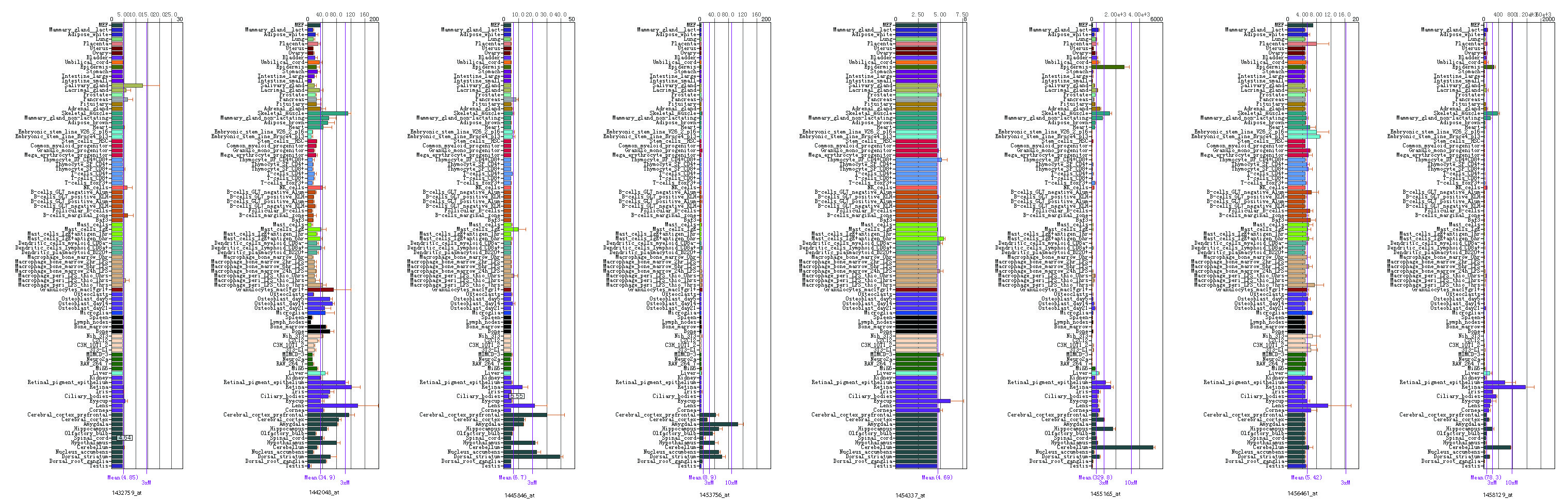

Supplement: Figure S4 — The distribution of expression across adult tissues (Mouse 430 2.0 array) for probes matched with putative embryonic brain lncRNAs. The expression information is taken from BioGPS server [65]. We obtain eight lncRNAs which are associated with non-redundant probes, of which seven probes are brain expressed and even brain-specific expressed. Even, four probes are highly expressed markedly in brain. Therefore, putative intergenic lncRNAs in embryonic brain are also expressed in adult brain related organs and tissues. (JPG) [file pone.0071152.s004.jpg]

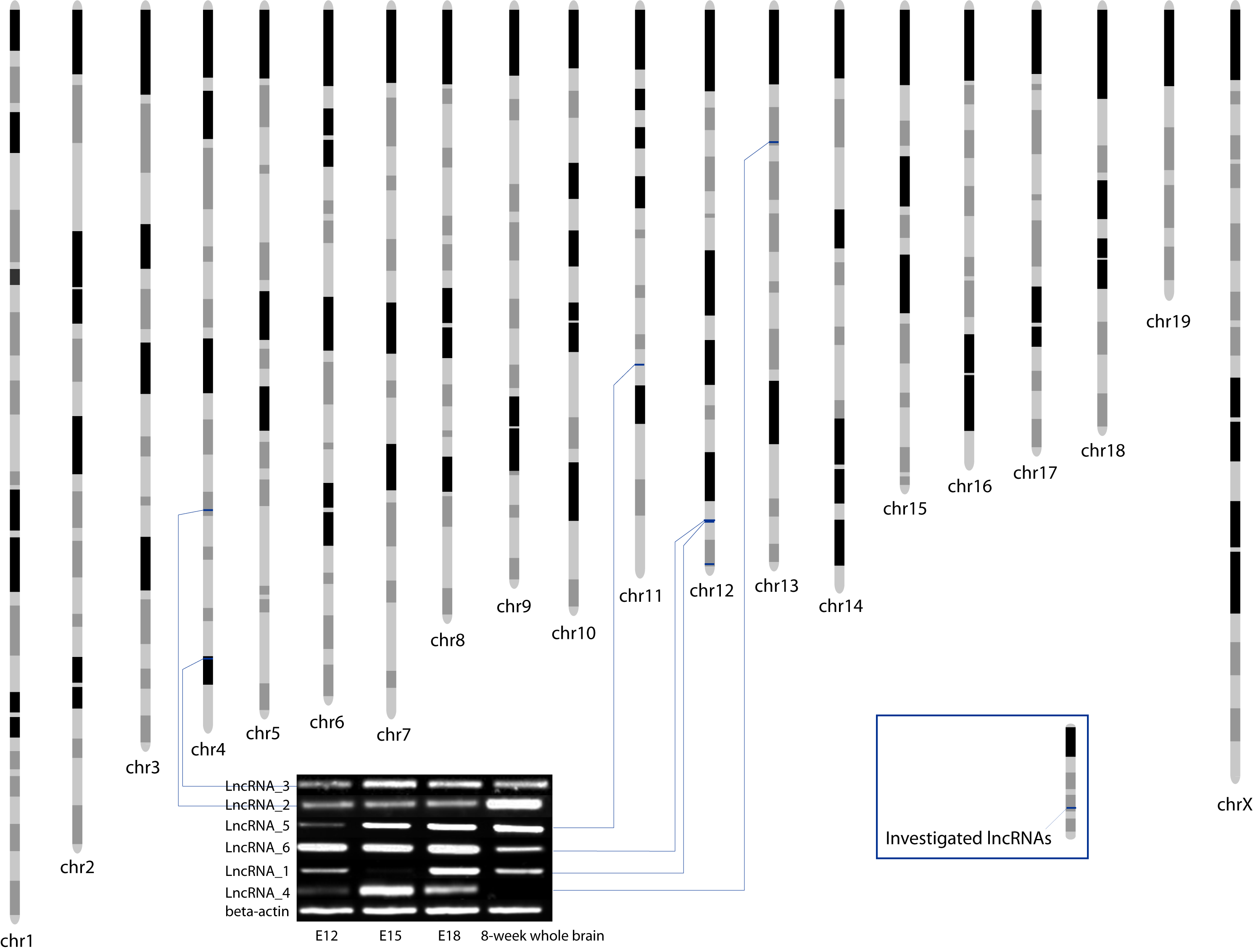

Supplement: Figure S5 — Chromosomal distribution of lncRNAs in the mouse genome and randomly chosen putative embryonic brain lncRNAs for exploring expression in adult whole brain. For each chromosome, the chromosomal coordinates start from top (0) to bottom. Six lncRNA candidates are marked by blue horizontal bars overlaid on chromosomes and are investigated by RT-PCR with four developmental time points (refer to Materials and Methods), while genomic details are in Table S3. LncRNA_x represents putative embryonic lncRNA ID in Table S3. (TIF) [file pone.0071152.s005.tif]
